# Interaction between HCMV pUL83 and human AIM2 disrupts the activation of the AIM2 inflammasome

**DOI:** 10.1186/s12985-016-0673-5

**Published:** 2017-02-20

**Authors:** Yuan Huang, Di Ma, Heyu Huang, Yuanyuan Lu, Yi Liao, Lingling Liu, Xinglou Liu, Feng Fang

**Affiliations:** 1 Department of Pediatrics, Tongji Hospital, Tongji Medical College, Huazhong University of Science and Technology, Wuhan, 430030 China; 2 Teaching and research office of pediatrics, Tongji hospital, Jiefang Road No. 1095, Qiaokou District, Wuhan, 430030 China

**Keywords:** HCMV, pUL83, AIM2 inflammasome, Immune evasion

## Abstract

**Background:**

AIM2, a cytosolic DNA sensor, plays an important role during infection caused by pathogens with double-stranded DNA; however, its role in human cytomegalovirus (HCMV) infection remains unclear. Previously, we showed an increase in AIM2 protein levels during the early stage of HCMV infection and a decrease 24 h post infection. Because HCMV has developed a variety of strategies to evade host immunity, we speculated that this decline might be attributed to a viral immune escape mechanism. The tegument protein pUL83 is an important immune evasion protein and several studies have reported that pUL83 binds to specific cellular proteins, such as AIM2-like receptor IFI16, to affect their functions. To determine whether pUL83 contributes to the variation in AIM2 levels during HCMV infection, we investigated the pUL83/AIM2 interaction and its impact on the AIM2 inflammasome activation.

**Methods:**

We constructed plasmids expressing recombinant pUL83 and AIM2 proteins for two-hybrid and chemiluminescence assays. Using co-immunoprecipitation and immunofluorescent co-localization, we confirmed the interaction of pUL83/AIM2 in THP-1–derived macrophages infected with HCMV AD169 strain. Furthermore, by investigating the expression and cleavage of inflammasome-associated proteins in recombinant HEK293T cells expressing AIM2, apoptosis-associated speck-like protein (ASC), pro-caspase-1 and pro-IL-1β, we evaluated the effect of pUL83 on the AIM2 inflammasome.

**Results:**

An interaction between pUL83 and AIM2 was detected in macrophages infected with HCMV as well as in transfected HEK293T cells. Moreover, transfection of the pUL83  expression vector into recombinant HEK293T cells stimulated by poly(dA:dT) resulted in reduced expression and activation of AIM2 inflammasome-associated proteins, compared with the absence of pUL83.

**Conclusions:**

Our data indicate that pUL83 interacts with AIM2 in the cytoplasm during the early stages of HCMV infection. The pUL83/AIM2 interaction deregulates the activation of AIM2 inflammasome. These findings reveal a new strategy of immune evasion developed by HCMV, which may facilitate latent infection.

## Background

Human cytomegalovirus (HCMV) is one of the most ubiquitous pathogens in the world. In immunocompetent individuals, HCMV infections usually progress to lifelong persistent latency after a short-term lytic infection, unaffected by the host immune system. HCMV has evolved multiple strategies to circumvent the innate and adaptive immune responses to establish such a long period of coexistence in the host [1–3]. The immune evasion is ascribed to the 230-kbp viral genome and enormous proteome [4].

pUL83 (also termed pp65) accounts for 15% of total virion protein [5] and is the most abundant tegument protein. It plays a role during cell entry and in the transcription of immediate-early (IE1 and IE2) genes [6, 7]. In addition to these roles in viral physiology, pUL83 is involved in immune evasion, which is pivotal during HCMV infection. For instance, pUL83 phosphorylates IE proteins to prevent immunological recognition of the virus [8, 9]. Interferon (IFN) levels in fibroblasts infected by the HCMV△pp65 strain, a mutant virus lacking the UL83 open reading frame (ORF), are higher than those in cells infected with wild-type virus. In contrast, over-expression of pUL83 partially blocks the IFN response, indicating that pUL83 is irredundant in suppressing the cellular IFN response to HCMV infection [10]. Moreover, pUL83 directly and specifically binds natural killer (NK)-activating receptor NKp30 to suppress the activation of NK cells [11].

Absent in melanoma 2 (AIM2) protein contains a C-terminal hematopoietic IFN-inducible nuclear (HIN) domain, which recognizes double-stranded (ds) DNA; and an N-terminal pyrin domain, which binds to apoptosis-associated speck-like protein (ASC) and subsequently recruits pro-caspase-1 for its auto-cleavage and proinflammatory cytokine maturation [12–17]. The AIM2 inflammasome is indispensable during certain infections [18]. Although its role in the immune response to HCMV remains unclear, multiple studies have provided indirect evidence for the possibility that AIM2 can recognize HCMV, as follows. (i) The HIN domain of AIM2 recognizes dsDNA through electrostatic interactions, irrespective of the DNA sequence and GC content, but in a length-dependent manner [17, 19]. (ii) Aim2, a murine homologue of AIM2, plays an important role in mouse cytomegalovirus (MCMV) infection [18]. (iii) Several researchers reported that HCMV infection induces the secretion of inflammatory cytokines such as interleukin (IL)-1β in serum of renal transplant recipients who developed a primary HCMV infection and IL-18 produced by HCMV-infected gingival fibroblasts [20, 21]. Even though the presence of Z-DNA binding protein 1 (ZBP1) was sufficient to enhance HCMV-stimulated transcription and secretion of IFN-β, its role in the release of IL-1β and IL-18 remains unconfirmed [22]. This suggests the existence of other immune pathways that activate these two cytokines during HCMV infection. Furthermore, Cristea et al. reported that HCMV pUL83 hijacks IFI16 to activate the major immediate early promoter (MIEP) through binding to the pyrin domain of IFI16 [7, 23]. Considering that AIM2 is in the same protein family as IFI16 and has a pyrin domain, we hypothesized that pUL83 is involved in the immune evasion of AIM2 inflammasome in a protein-protein interaction-dependent manner. Verification of this hypothesis comprises the aim of this study. We analyzed the interaction between pUL83 and AIM2 in recombinant HEK293T cells using two-hybrid and chemiluminescence assays. We also used co-immunoprecipitation and immunofluorescent co-localization experiments to study the interaction in HCMV-infected THP-1–derived macrophages. Furthermore, we evaluated the impact of pUL83 on AIM2 inflammasome activation in recombinant HEK293T cells expressing AIM2, ASC, pro-caspase-1, and pro-IL1β.

## Methods

### Cells and virus

MRC-5 and HEK293T cells were sustained in Dulbecco’s modified Eagle medium (DMEM) (Gibco) contained 10% newborn calf serum (Gibco). THP-1 cells were cultured in RPMI 1640 medium contained 10% fetal bovine serum (Gibco). HCMV AD169 strain was propagated in MRC-5 cells and stored in liquid nitrogen.

### Competent cells and plasmid vectors

Stellar Competent Cells (Clontech) were stored at −80 °C. Luria-Bertani (LB) medium (yeast extract, peptone, NaCl), with or without agar, was proceeded autoclaving before adding ampicillin (50 μg/ml) or kanamycin (30 μg/ml), and stored at 4 °C. pM GAL4-BD Cloning Vector (pM, 3.5 kbp), encoding the DNA binding domain (BD) of GAL4, pVP16 AD Cloning Vector (pVP16, 3.3 kbp), encoding activating domain (AD) of GAL4, and pG5SEAP Reporter Vector (pG5SEAP), containing secreted alkaline phosphatase (SEAP) gene with an upstream activating sequence (UAS) were contained in Matchmaker™ Mammalian Assay Kit (cat. 630305) purchased from Clontech, as well as pM3-VP16 Positive Control Vector (pM3-VP16), pM-53, pVP16-T and pVP16-CP. All vectors contain ampicillin resistance gene. pDsRed2-N1, containing kanamycin resistance gene, was used to recombine AIM2 inflammasome proteins expression vectors.

### Reagents

Restriction endonuclease (EcoRI, SalIand BamHI), PrimeScript™ II 1st strand cDNA Synthesis Kit and PrimeSTAR® HSDNA Polymerase were obtained from Takara. Gel Extraction Kit, Plasmid Extraction Kit and Endo-free plasmid kit were from Omega. In-Fusion® HD Cloning Kit (Clontech, cat. 639648) was used for inserting desired genes into vectors. CalPhos™ Mammalian Transfection Kit (Clontech, cat. 631312) was for transfecting reconstructed plasmids into mammalian cells, and Great EscAPe SEAP Chemiluminescence Detection Kit (Clontech, Cat. 631701) was bought for assaying SEAP. Phorbol myristate acetate (PMA) (Sigma, USA) was applied for THP-1 cell differentiation. Protein A/G bead was from Thermo Fisher Scientific, pUL83 antibody (Abcam, cat. ab6503), AIM2 antibody (CST, cat. D5X7K), ASC (Santa Cruz, cat. sc-30153), caspase-1 antibody (Santa Cruz, cat. sc-515), IL-1β antibody (Biovision, cat. 5128) and fluorescent tagged second antibodies were used at recommended concentrations. 4′, 6-diamidino-2-phenylindole (DAPI) was used at 2 μg/mL.

### Primer design

UL83 ORF (1686 bp, GenBank: KJ743149.1), AIM2 mRNA (1032 bp, NCBI Reference Sequence: NM_004833.1), ASC mRNA (588 bp, GenBank: AB023416.2), Caspase-1 mRNA (1209 bp, NCBI Reference Sequence: NM_012762.2) and IL-1β mRNA (810 bp, NCBI Reference Sequence: NM_000576.2) were picked as templates to design primers using Clontech online prime design tool for In-Fusion. According to the user manual of In-Fusion clone, the 5’ end of each primer was deliberately added 15 bases that are homologous to 15 bases at one end of the vector (italics) and restriction enzyme sites (bold italics) were fully retained (Table 1).

**Table 1:** Primers for genes of interest

UL83	forward	5′-*TGTATCGCCG* ***GAATTC***TCAACCTCGGTGCTTTTTG-3′
	reverse	5′-*CTGCAGACGC* ***GTCGAC***ATGGAGTCGCGCGGTCGC-3′
AIM2	forward	5′-*GTACGGTGGG* ***GAATTC***GGAGGCTGATCCCAAAGTTGT-3′
	reverse	5′-*ACGCGTCGAC* ***GGATCC***TGCTGCTTAGACCAGTTGGC-3′
ASC	forward	5′-*CTCAAGCTTC* ***GAATTC***ATGGGGCGCGCGCGCGAC-3′
	reverse	5′-*CCGCGGTACC* ***GTCGAC***TCAGCTCCGCTCCAGGTC-3′
caspase-1	forward	5′-*CTCAAGCTTC* ***GAATTC***ATGGCCGACAAGGTCCTG-3′
	reverse	5′-*CCGCGGTACC* ***GTCGAC***TTAATGTCCTGGGAAGAGG-3′
IL-1β	forward	5′-*CTCAAGCTTC* ***GAATTC***ATGGCAGAAGTACCTGAGC-3′
	reverse	5′-*CCGCGGTACC* ***GTCGAC***TTAGGAAGACACAAATTGCAT-3′

Italics: 15 bases homologous to one end of vector. Bold italics: restriction enzyme sites

### Construction of recombinants plasmids

#### Linearization and purification of vectors

pM and pDsRed2-N1 were digested by EcoRI and SalI overnight at 37 °C, while pVP16 was digested by EcoRI and BamHI overnight at 30 °C. Agarose gel electrophoresis (AGE) isolated fragments were subsequently purified by Gel Extraction Kit according to the user guide.

#### Reverse-transcription polymerase chain reaction (RT-PCR) and purification of fragments

HCMV AD169 infected THP-1–derived macrophages were collected to extract total RNA and synthesize cDNA, which used as templates to amplify desired genes using indicated primers.

#### In-Fusion cloning

ORFs of UL83 and AIM2 were respectively inserted into linearized pM and pVP16 vectors; ORFs of ASC, caspase-1 and IL-1β were inserted into linearized pDsRed2 vectors separately according to In-Fusion® HD Cloning Kit user manual. Reaction mixtures were then transformed to competent cells for incubation on resistant medium plates.

#### The confirmation of recombinants

Monoclone was proliferated in LB liquid medium before plasmid extraction. Recombinants were roughly identified by double enzyme digestion and PCR. pM-UL83 was digested with EcoRI and SalI at 37 °C and pVP-AIM2 was digested with EcoRI and BamHI at 30 °C. Undigested recombinant plasmids were used as templates for the amplification of desired genes. Digested and PCR products were subjected to AGE. Sequencing confirmation was then applied. Plasmids were extracted with Endo-free plasmid kit and transfected into HEK293T cells for 72 h and the expression of recombinants were detected by SDS-PAGE (12%).

### Two-hybrid and chemiluminescence assay

HEK293T cells were seeded into 10-cm petri dishes and incubated for 24 h. Then the medium was replaced by fresh DMEM complete medium and incubated for another 2 h or more until the cells achieve 70% confluence. Using the calcium phosphate transfection method, plasmids were transfected into HEK293T cells. 8 h later, calcium phosphate-containing medium was exchanged by DMEM complete medium and cells were incubated for 72 h. Supernatant of each dish was centrifuged to discard cell debris and then subjected to SEAP detection by chemiluminescence at 405 nm. Statistical data were analyzed by *T*-test.

### Co-immunoprecipitation

Cells were harvested and lysed with cold protein lysis buffer (50 mM HEPES, pH 7.4, 250 mM NaCl, 0.1% NP-40, 2 mM EDTA, 10% glycerol, protease inhibitors cocktail) for 30 min. Then centrifuged at 12000 rpm for 10 min at 4 °C. Supernatant of cell lysates were transferred into new tubes and mixed with primary antibodies and incubated at 4 °C with gentle agitation overnight. Then protein A/G beads was added to capture antigen-antibody complex, which subsequently proceeded heat denaturing and immunoblotting.

### Immunoblotting

Cells lysates were prepared as mentioned above. Heat denatured cell lysates were then subjected to SDS-PAGE and transferred to PVDF membranes. The membranes were blocked with 5% skim milk and incubated with primary antibodies overnight at 4 °C, and subsequently incubated with horseradish peroxidase (HRP)-conjugated secondary antibodies before processing exposure.

### Immunofluorescent

Cells were washed twice with cold phosphate buffer saline (PBS), and fixed with 4% paraformaldehyde for 10 min. Then appropriate amount of 0.3% TritonX-100 was added. Normal non-immune serum was used to block non-specific epitopes. Cells were incubate with specific primary antibodies overnight at 4 °C, and subsequently incubated with fluorescent labelling secondary antibodies before observing with fluorescence microscope.

### Statistical analysis

The means of triplicate samples were compared using *T*-test statistical method with GraphPad Prism software (GraphPad Software, USA). A *P* value of <0.01 was considered as statistically significant.

## Results

### Plasmids for expression of recombinant pUL83 and AIM2 proteins

MRC-5 cells were infected with HCMV AD169 strain for 2 d, until pUL83 was highly expressed [24]. The cells were then collected and UL83 and AIM2 genes were amplified by RT-PCR. The genes were used as templates in subsequent in-fusion cloning.

**Fig. 1:**
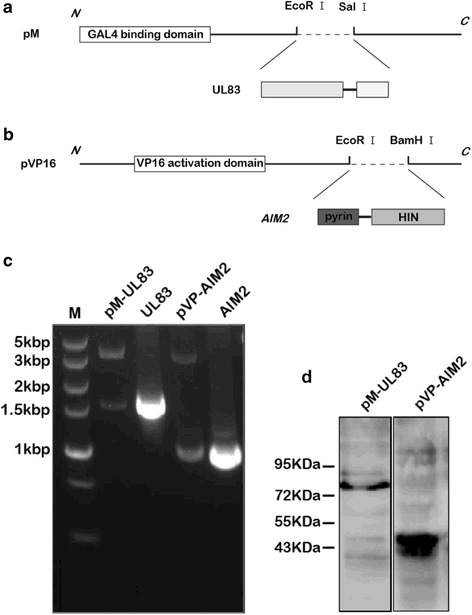
Construction and expression of recombinant UL83 and AIM2 proteins. **a** UL83 ORF (1686 bp) was cloned into the MCS of the pM vector for the expression of a fusion of a bait protein (pUL83 herein) with Gal4 DNA BD (147 aa). **b** AIM2 ORF (1024 bp) was inserted into pVP16 vector to express recombinant AIM2 (the prey protein) fused to VP16 AD (45 aa). **c** Plasmids digested by EcoRI and SalI or EcoRI and BamHI and PCR products were analyzed by agarose gel electrophoresis. The sizes of the plasmid digestion and PCR products were as anticipated. **d** Verified recombinants were used to transfect HEK293T cells for 72 h. The expression of target proteins was assessed by SDS-PAGE with specific antibodies against pUL83 and AIM2. The expected recombinant protein sizes were 81 kDa (BD-pUL83) and 44 kDa (AD-AIM2)

The pM GAL4-BD cloning vector was used to construct the pM-UL83 vector, where the UL83 ORF was inserted into the multiple cloning site (MCS) (Fig. 1a). The AIM2 ORF was cloned into the pVP16 AD cloning vector to fuse AIM2 with AD (Fig. 1b). The recombinant plasmids pM-UL83 and pVP-AIM2 were first verified by restriction endonuclease cleavage and PCR (Fig. 1c). Further nucleotide sequencing revealed 100% sequence identity with the UL83 and AIM2 genes. Good expression of the recombinant pUL83 and AIM2 proteins were observed in HEK293T cells (Fig. 1d).

### Recombinant pUL83 and AIM2 proteins interact with each other in mammalian cells

**Fig. 2:**
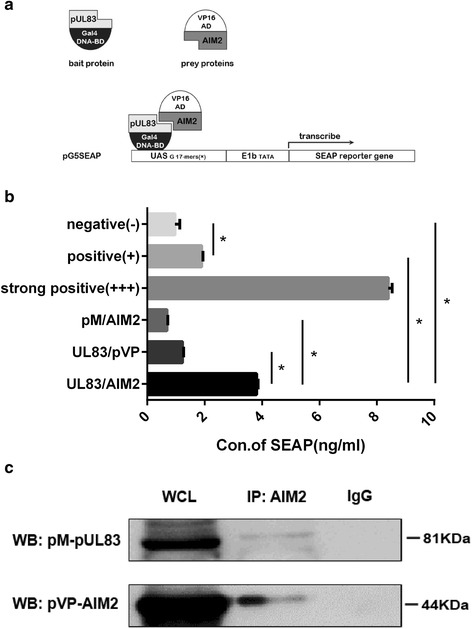
Detection of the interaction between pUL83 and AIM2. **a** Schematic diagram of a two-hybrid experiment, adapted from the Matchmaker Mammalian Assay Kit. **b** Plasmids encoding recombinant pUL83 and AIM2 proteins were used together with pG5SEAP to co-transfect HEK293T cells for 72 h. Supernatants were then collected and SEAP levels were detected by chemiluminescence at 405 nm. The experiment was repeated three times. Statistical data were analyzed using the *t*-test. **c** Plasmids encoding recombinant pUL83 and AIM2 were used to transfect HEK293T cells for 72 h. Cells were harvested and lysed with protein lysis buffer, and whole cell lysates were immunoblotted using specific antibodies against pUL83 and AIM2, or immunoprecipitated with the anti-AIM2 antibody and then detected using the anti-pUL83 antibody. IgG was used as a negative control. Data from one representative experiment out of three are presented as the mean ± SD. * *P* < 0.01. WCL: whole cell lysates

We detected an increase in AIM2 protein levels in THP-1 − derived macrophages 3 h post HCMV infection, which gradually increased up to 12 h. However, the level was lower at 24 h than at 12 h for unknown reasons (unpublished data). To investigate whether the attenuation of the AIM2 inflammasome was linked to HCMV pUL83, we first determined the interaction between pUL83 and AIM2 using two-hybrid system. The main principle of the two-hybrid system is that BD and AD will act together as a transcriptional activator if they are tethered in space, even if they belong to separate proteins [25, 26]. Accordingly, an interaction between pUL83 and AIM2 should result in co-localization of DNA-BD and AD, leading to transcription of the *SEAP* reporter gene from pG5SEAP (Fig. 2a). We used pM-UL83, pVP-AIM2, and pG5SEAP to co-transfect HEK293T cells, henceforth referred to as pM-UL83/pVP-AIM2. Several experimental controls were also prepared (Table 2). pM3-VP16 is a strong positive control expressing a fusion of GAL4 DNA-BD to the VP16 AD; pM-53 expresses a fusion of GAL4 DNA-BD to the mouse p53 protein; and pVP16-T expresses a fusion of VP16 AD to the SV40 large T-antigen, which is known to interact with p53 protein. pVP16-CP expresses a fusion of the VP16 AD to a viral coat protein, which does not interact with p53. Co-transfection of pM-53 and pVP16-T was used as a weak positive control, while co-transfection of pM-53 and pVP16-CP was negative control. Culture supernatants were collected 72 h post-transfection to assess secreted SEAP levels. As shown in Fig. 2b, pM-UL83/pVP-AIM2 released more SEAP into the culture supernatants than the weak positive control and some other controls (*P* < 0.01), but less SEAP than the strong positive control. This suggested a possible interaction between pUL83 and AIM2. Negative control and auto-activation detection groups produced very low levels of SEAP, indicating that pM and pVP16 had no transcriptional activity by themselves.

**Table 2:** Experimental and control groups

Experimental	pM-UL83/pVP-AIM2
Bait protein control^△^	pM-UL83/pVP16
Prey protein control^△^	pM/pVP-AIM2
Strong positive control	pM3-VP16
Weak positive control	pM-53/pVP16-T
Negative control	pM-53/pVP16-CP
Basal control^▲^	pM/pVP16
Untransfected control^a^	none

△: These controls aim at excluding the possibility of non-carrier self-activation, ^▲^: This control provides the basal expression level of SEAP. ^a^: This control reveals the background SEAP signal

We next verified these results by performing co-immunoprecipitation experiments. pM-UL83 and pVP-AIM2 were co-transfected into HEK293T cells. Cell lysates were used in immunoprecipitation experiments with anti-AIM2 antibodies, and the antigen-antibody complex was detected by immunoblotting with anti-pUL83 antibodies. A moderate band at the expected size of recombinant pUL83 (81 kDa) was detected (Fig. 2c). These preliminary analyses support the interaction between pUL83 and AIM2.

### pUL83 associates with AIM2 in THP-1 − derived macrophages infected with HCMV

**Fig. 3:**
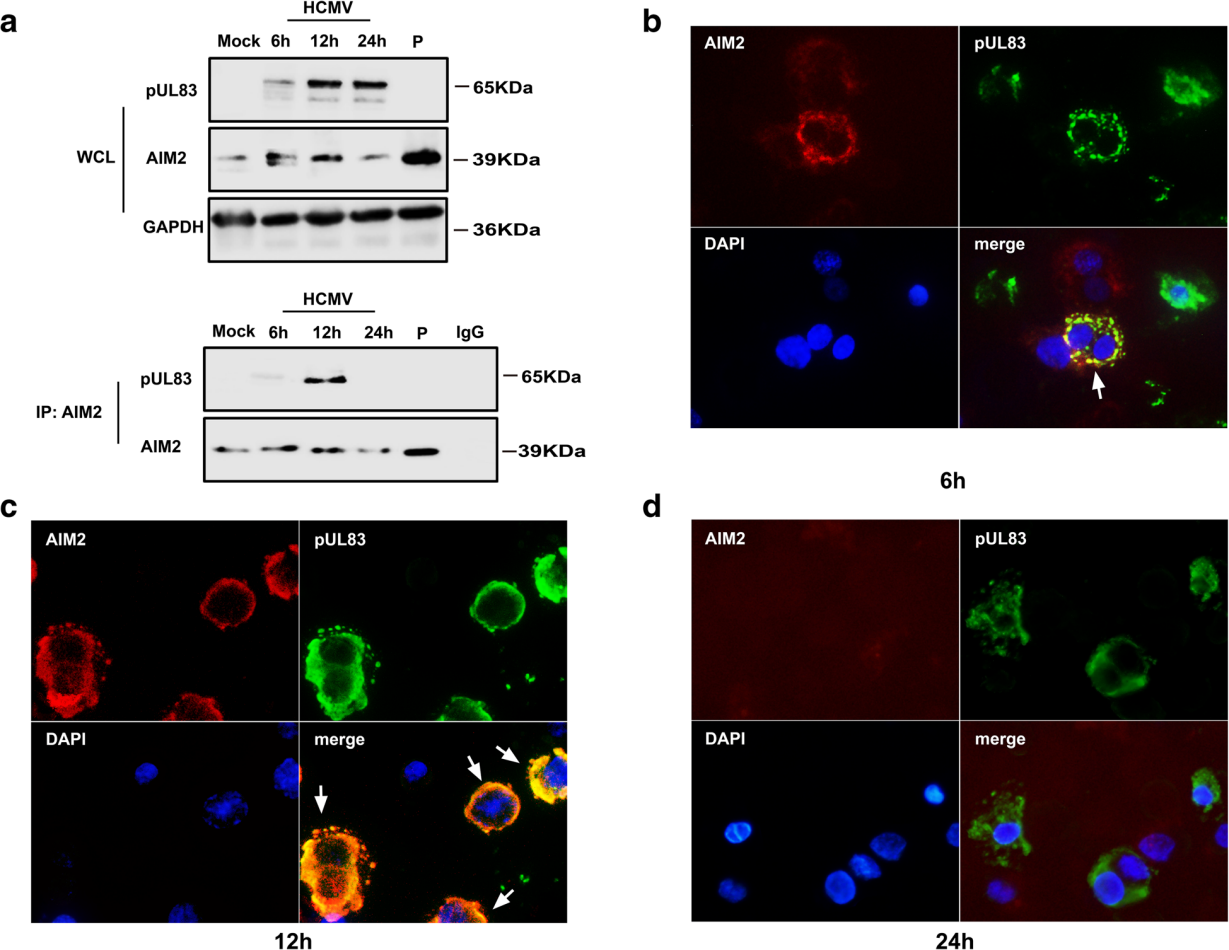
Detection of the pUL83/AIM2 interaction in HCMV-infected cells. **a** THP-1 cells were stimulated with PMA (100 ng/mL) to induce cellular differentiation. They were then mock-infected or infected with the HCMV AD169 strain for 6 h, 12 h, or 24 h, or transfected with poly(dA:dT). The cells were harvested and lysed and whole-cell lysates were immunoblotted using specific antibodies against pUL83 and AIM2, or immunoprecipitated with the anti-AIM2 antibody and then detected with anti-pUL83 and anti-AIM2 antibodies. **b–d** The infected cells were washed and fixed at the indicated time points. Specific antibodies against pUL83 and AIM2 were added and then conjugated with fluorescently tagged secondary antibodies. Cell nuclei were stained with DAPI. P: poly(dA:dT). WCL: whole cell lysates. *Red*, AIM2; *green*, pUL83; DAPI (*blue*), nuclei

To determine whether a *bona fide* interaction between pUL83 and AIM2 occurs in vivo, we further probed this interaction in HCMV-infected cells. THP-1–derived macrophages were mock-infected or infected with the HCMV AD169 strain for 6 h, 12 h, and 24 h. The identical cells transfected with poly(dA:dT) were used as a positive control for AIM2 or a negative control for pUL83. Cell lysates were analyzed by immunoblotting and immunoprecipitation. pUL83 was detected in whole cell lysates at all of the indicated times. Expression of AIM2 was prominent 6 h and 12 h post infection, but it was only weakly apparent 24 h post infection, comparable with the mock-infected control. THP-1 *−* derived cells transfected with poly(dA:dT) expressed AIM2 but not pUL83 (Fig. 3a, WCL fraction). Following immunoprecipitation with the anti-AIM2 antibody, cell lysates were also assayed by immunoblotting. A moderate pUL83-specific band was observed 6 h post infection, and a more distinct band was seen 12 h post infection (Fig. 3a, IP: AIM2 fraction). This indicated that pUL83 interacts with AIM2 6 h and 12 h post infection.

We verified these results by performing immunofluorescence experiments. THP-1 − derived macrophages were infected with the HCMV AD169 strain for the indicated time periods. We observed that pUL83 (green signal) and AIM2 (red signal) co-localized in the cytoplasm at 6 h and 12 h post infection (Fig. 3b, c, white arrow), whereas the AIM2 signal weakened at 24 h post infection (Fig. 3d). This was consistent with the results of immunoblotting and immunoprecipitation experiments. In summary, our data suggest that pUL83 interacts with AIM2 in HCMV-infected macrophages at the early infection stage.

### pUL83/AIM2 complex results in declining abundance of inflammasome proteins

**Fig. 4:**
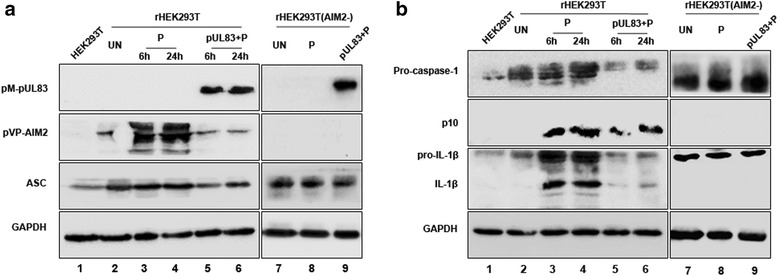
The effect of the pUL83/AIM2 complex on the AIM2 inflammasome. ASC, pro-caspase-1, and pro-IL-1β ORFs were inserted into pDsRed2-N1 expression vectors. The recombinant vectors with or without the pVP-AIM2 vector were used to co-transfect HEK293T cells transiently for 72 h. The resultant cells were named rHEK293T or rHEK293T (AIM2-). Protein expression of these cells (**a** and **b**, line 2 and 7) as well as that of wild HEK293T cells (**a** and **b**, line 1) was determined by immunoblotting. Poly(dA:dT) was used to transfect rHEK293T cells for 6 h and 24 h or transfect rHEK293T (AIM2-) cells for 6 h to activate the AIM2 inflammasome, and the expression and activation of inflammasome proteins were then determined (**a** and **b**, lines 3, 4 and 8). The UL83 expression vector was used to transfect rHEK293T cells, which were then stimulated with poly(dA:dT); the inflammasome proteins were then detected (**a** and **b**, lines 5, 6 and 9). P: poly(dA:dT). p10: the activated form of caspase-1

Because the AIM2 inflammasome plays an important role in the host defense against infection, we investigated whether the association between pUL83 and AIM2 affects the subsequent assembly of the inflammasome and IL-1β activation. ASC, pro-caspase-1, and pro-IL-1β expression vectors were constructed and used with pVP-AIM2 to co-transfect HEK293T cells. The transient transfectants were named rHEK293T. Poly(dA:dT) was used to transfect rHEK293T to stimulate the activation of the AIM2 inflammasome. To study the impact of pUL83 on the activation of the AIM2 inflammasome, rHEK293T cells were transfected with the pUL83 expression vector prior to poly(dA:dT) stimulation. pUL83 and AIM2 inflammasome-associated proteins were assayed by immunoblotting. AIM2, ASC, pro-caspase-1, and pro-IL-1β were highly expressed in rHEK293T cells (Fig. 4a, b, line 2). Furthermore, the expression of AIM2, pro-caspase-1, and pro-IL-1β increased both 6 h and 24 h after stimulation with poly(dA:dT). The activated form of caspase-1, p10, and cleaved IL-1β were also detected (Fig. 4a, b, line 3, 4), implying that the AIM2 inflammasome was activated. In contrast, pUL83-expressing rHEK293T cells seemed unresponsive to poly (dA:dT) because the expression of AIM2, pro-caspase-1, and pro-IL1β was reduced, with that of p10 slightly reduced and that of IL-1β dramatically reduced (Fig. 4a, b, line 5, 6) upon poly(dA:dT) stimulation.

To determine whether the reduction of protein levels was caused by the interaction of pUL83 and AIM2 or pUL83 alone, we retested the above experiments in recombinant HEK293T cells expressing ASC, pro-caspase-1, and pro-IL1β, but no AIM2 (Fig. 4a, b, line 7–9). The protein abundance of ASC, pro-caspase-1, and pro-IL-1β was not altered by poly(dA:dT) or pUL83 in AIM2-deficient recombinant HEK293T cells. Additionally, no cleaved caspase-1 or mature IL-1β was detected. These results collectively indicated that the pUL83/AIM2 complex mediated the attenuation of AIM2 inflammasome proteins and subsequently reduced the cleavage of caspase-1 and maturation of IL-1β.

## Discussion

pUL83 is the most abundant tegument protein and is involved in various biochemical processes in infected cells [27]. Its ability to engineer immune escape is especially worth noting. Interestingly, even though pUL83 is dispensable for viral growth in human fibroblasts [28], the proliferation of an HCMV variant lacking pUL83 is seriously compromised in monocyte-derived macrophages [27] that constitutively express AIM2 [29]. The different requirements for pUL83 may reflect distinct responses of different cell lines to HCMV. In our previous study involving THP-1 − derived macrophages, we observed an increase in AIM2 levels at the early stage of HCMV infection; however, 24 h post infection, they returned to the basal level (unpublished data). We proposed that such attenuation of AIM2 inflammasome 24 h post HCMV infection was linked to pUL83. To investigate the relationship between pUL83 and AIM2 in detail, we performed a two-hybrid assay to assess their putative protein-protein interaction in vitro. We successfully constructed pUL83 and AIM2 expression vectors, where the respective proteins were fused with GAL4 BD and AD. These vectors were then used to transfect HEK293T cells. As anticipated, we detected the expression of the reporter gene. This result was confirmed in HCMV-infected THP-1–derived macrophages. We observed that the pUL83/AIM2 complex was indeed formed and localized in the cytoplasm during the early stage of infection, particularly 12 h post infection, but not 24 h post infection. In addition, AIM2 protein levels decreased 24 h post infection, which reinforced our unpublished preliminary observations. Because the AIM2 inflammasome plays an important role in the immune response, we wondered whether the pUL83/AIM2 interaction constitutes one of the immune evasion strategies developed by HCMV, i.e., suppression of the function of the AIM2 inflammasome. Using a published approach [30], we constructed recombinant HEK293T (rHEK293T) cells expressing AIM2, ASC, pro-caspase-1, and pro-IL1β, and examined the effect of pUL83 on the AIM2 inflammasome in these cells. Compared with poly(dA:dT)-stimulated rHEK293T cells, where a high level of inflammasome activation was maintained, AIM2 protein level, as well as pro-caspase-1 and pro-IL-1β protein levels, were dramatically reduced when pUL83 was expressed, with a slight decrease in ASC protein level. We next ruled out the independent effect of pUL83 on the indicated proteins (except AIM2). We therefore conclude that the pUL83/AIM2 interaction is responsible for the attenuation of AIM2 inflammasome proteins, followed by reduced cleavage of caspase-1 and IL-1β. Because these proteins are constitutively expressed in rHEK293T cells, we surmise that the decline in protein abundance results from increased protein degradation rather than reduced gene expression. According to our results, caspase-1 activated form, p10, and IL-1β were still weakly detected in rHEK293T cells in the presence of pUL83, indicating that rather than preventing the assembly of the AIM2 inflammasome, pUL83 facilitates the degradation of the assembled AIM2 inflammasome through binding to AIM2. Autophagy is a cell homeostatic process that mediates the degradation of cytosolic protein aggregates [31]. An increasing number of studies has shown involvement of autophagy in the regulation of the inflammasome [32, 33]. For instance, autophagy controls the production of IL-1β through sequestering and targeting of pro-IL-1β for lysosomal degradation [34]. In addition, autophagocytosis-deficient Atg16−/− mice accumulated excessive IL-1β in response to LPS [35]. Recently, Nurmi et al. found that intraperitoneal administration of a hemin derivative depleted ASC in mice macrophages, which was attributed to the autophagy pathway [36]. Moreover, a previous study showed that the AIM2 inflammasome could trigger and in turn be degraded by autophagy [37]. We therefore propose that the pUL83/AIM2 complex might enhance the autophagy pathway and accelerate the degradation of inflammasome proteins.

## Conclusion

In summary, our data indicate that the HCMV tegument protein pUL83 binds to cellular AIM2, which partially contributes to the attenuation of the AIM2 inflammasome proteins 24 h post HCMV infection and reduced activation of caspase-1 and IL-1β. This effect of the pUL83/AIM2 interaction may facilitate the latency of HCMV, hence informing the treatment of latent HCMV infections. However, our data are based on in vitro models of infection and should be verified in further experiments. The proposed biological significance of the pUL83/AIM2 interaction should be investigated in depth in an in vivo infection model, by either overexpressing or deleting the UL83 gene. Other experimental approaches such as fluorescence resonance energy transfer (FRET) and high-resolution electron microscopy should be used to obtain physical evidence of this interaction.

## Abbreviations

AD: Activating domain
AGE: Agarose gel electrophoresis
AIM2: Absent in melanoma 2
ASC: Apoptosis-associated speck-like
BD: Biding domain
DAPI: 4′,6-diamidino-2-phenylindole
dsDNA: Double-stranded DNA
HCMV: Human cytomegalovirus
HIN: Hematopoietic IFN-inducible nuclear
IE: Immediate-early
IFN: Interferon
IL: Interleukin
IRF1: Interferon regulatory factor 1
MCMV: Mouse cytomegalovirus
MCS: Multiple cloning site
MIEP: Major immediate early promoter
NK: Natural killer
ORF: Open reading frame
PBS: Phosphate buffer saline
PMA: Phorbol myristate acetate
RT-PCR: Reverse-transcription polymerase chain reaction
SDS-PAGE: Sodium dodecyl sulfate polyacrylamide gel electrophoresis
SEAP: Secreted embryonic alkaline phosphatase
UAS: Upstream activating sequence
ZBP1: Z-DNA binding protein 1
